# Effectors involved in fungal–fungal interaction lead to a rare phenomenon of hyperbiotrophy in the tritrophic system biocontrol agent–powdery mildew–plant

**DOI:** 10.1111/nph.14851

**Published:** 2017-10-18

**Authors:** Joan Laur, Gowsica Bojarajan Ramakrishnan, Caroline Labbé, François Lefebvre, Pietro D. Spanu, Richard R. Bélanger

**Affiliations:** ^1^ Département de Phytologie Université Laval Québec QC Canada G1V 0A6; ^2^ Department of Life Sciences Imperial College London South Kensington Campus London SW7 2AZ UK

**Keywords:** biocontrol agent, effectors, haustorium, hyperbiotrophy, powdery mildew, *Pseudozyma flocculosa*, tritrophic interaction, Ustilaginales

## Abstract

- Tritrophic interactions involving a biocontrol agent, a pathogen and a plant have been analyzed predominantly from the perspective of the biocontrol agent. We have conducted the first comprehensive transcriptomic analysis of all three organisms in an effort to understand the elusive properties of *Pseudozyma flocculosa* in the context of its biocontrol activity against *Blumeria graminis* f.sp. *hordei* as it parasitizes *Hordeum vulgare*.
- After inoculation of *P. flocculosa*, the tripartite interaction was monitored over time and samples collected for scanning electron microscopy and RNA sequencing.
- Based on our observations, *P. flocculosa* indirectly parasitizes barley, albeit transiently, by diverting nutrients extracted by *B. graminis* from barley leaves through a process involving unique effectors. This brings novel evidence that such molecules can also influence fungal–fungal interactions. Their release is synchronized with a higher expression of powdery mildew haustorial effectors, a sharp decline in the photosynthetic machinery of barley and a developmental peak in *P. flocculosa*. The interaction culminates with a collapse of *B. graminis* haustoria, thereby stopping *P. flocculosa* growth, as barley plants show higher metabolic activity.
- To conclude, our study has uncovered a complex and intricate phenomenon, described here as hyperbiotrophy, only achievable through the conjugated action of the three protagonists.

## Introduction

Powdery mildew causes important losses to a wide range of agricultural and horticultural plants. Despite being recognized as one disease, over 650 powdery mildew species are known to occur on nearly 10 000 host species (Glawe, 2008). Powdery mildew fungi belong to the Erysiphales within the Ascomycota and are obligate biotrophic parasites. To establish infection, they depend on living cells where they form a specialized feeding structure, the haustorium, which invaginates the plasma membrane of the host epidermal cell. The interaction between the haustorium and the plant cell is tightly regulated so that the fungus can obtain resources from the plant without killing it (Chaudhari *et al*., 2014). Diversion of nutrients from the host to powdery mildew fungus leads to the production of mycelium, conidiophores and conidia, thus maintaining the development and asexual reproduction of the fungus. For the plant, the infection results in reduced photosynthesis, slower development and lower productivity.

The economic impact of powdery mildews has driven research in many areas and possibly none more than in the field of genetic control. The pioneering work of Moseman *et al*. (1964) was the first to investigate gene‐for‐gene interactions between isolates of cereal powdery mildew (*Blumeria graminis*) and varieties of barley and wheat. As a result, barley powdery mildew (*B. graminis* (DC.) Speer f. sp. *hordei* Marchal; *B. graminis*) is currently one of the best‐studied fungal diseases of plants in terms of the genetics of the host–pathogen interactions. Moreover, the *B. graminis* genome was the first to be released among powdery mildew species (Spanu *et al*., 2010), which led to the annotation of 6469 genes from which 491 were identified as candidate secreted effector proteins (CSEPs) (Pedersen *et al*., 2012). As in many plant–pathogen interactions, effector proteins are involved in disease development by, among other functions, suppressing host defense responses and affecting the host metabolism for the purpose of nutrient acquisition by the pathogen (Chaudhari *et al*., 2014). In spite of the overwhelming evidence linking CSEPs to the virulence arsenal of plant pathogens, very little is known about their functionality (Sonah *et al*., 2016). Out of the 491 putative CSEPs identified *in silico* in *B. graminis*, recent efforts have so far directly associated about a dozen candidate effectors to the virulence/aggressiveness of the pathogen (Pliego *et al*., 2013; Ahmed *et al*., 2015, 2016; Whigham *et al*., 2015).

Although *B. graminis* is widely recognized as a study model for powdery mildews, it is clear that its gene‐for‐gene interaction with barley is unique, this notion being reinforced by the fact that all 491 CSEPs are found only in *Blumeria* spp. Moreover, the majority of resistance genes that can be exploited in barley for powdery mildew control do not confer protection in other powdery mildew–plant interactions. For this reason, other research efforts have concentrated on the development of control methods that were more generic against powdery mildews. With growing concerns over the environmental impact of fungicides, an important body of work has concentrated on biological approaches. Many efforts have been directed toward the identification/isolation of natural enemies of powdery mildews that could be exploited as biocontrol agents (BCA) (Bélanger & Labbé, 2002; Kiss *et al*., 2010). Among the fungi and bacteria that have been reported to display an antagonistic activity against the Erysiphales, *Pseudozyma flocculosa* is arguably the most intriguing.

*Pseudozyma flocculosa* was first discovered and described in 1987 as an epiphytic yeast on powdery mildew‐infected clover leaves (Traquair *et al*., 1988). It was subsequently found to be a powerful antagonist of powdery mildews and its activity appears to extend to all and only members of the Erysiphales (Bélanger *et al*., 2012). Over the years, considerable resources have been invested in trying to decipher how *P. flocculosa* could specifically attack powdery mildews based on the principle that BCAs exert their activity through the manifestation of competition, parasitism, antibiosis or induced resistance (Whipps, 2001). In the case of *P. flocculosa*,* in vitro* bioassays, electron microscopy studies and chemical analyses have all pointed to a single mode of action: antibiosis. This conclusion was reinforced by the characterization and purification of an active molecule, flocculosin, that displayed powerful antimicrobial activity (Cheng *et al*., 2003; Mimee *et al*., 2005, 2009). This was further supported by the discovery of a complex gene cluster regulating the synthesis of flocculosin (Teichmann *et al*., 2011b). Intriguingly, flocculosin is nearly identical to ustilagic acid – a compound produced by *Ustilago maydis* under the control of a similar gene cluster (Teichmann *et al*., 2007, 2011a). However, the latter discovery, although confirming the classification of *P. flocculosa* within the Ustilaginales and its close link to smut pathogens, raised questions about the role of flocculosin in the biocontrol activity. If flocculosin indeed confers biocontrol activity against powdery mildews, what is the function of ustilagic acid in *U. maydis* considering that the fungus is a plant pathogen and does not act as BCA? In an effort to relate the production of flocculosin (or ustilagic acid) to biocontrol, Clément‐Mathieu *et al*. (2008) and Hammami *et al*. (2011) tested and compared *P. flocculosa, U. maydis* and other species of *Pseudozyma* with or without the ability to produce glycolipids. In all bioassays, only *P. flocculosa* was capable of antagonizing powdery mildews. The authors concluded that the biocontrol specificity of *P. flocculosa* could not be attributed to its ability to produce flocculosin alone, given that other similar organisms producing the same glycolipids were incapable of biocontrol. Rather, the results suggested that the particular properties of *P. flocculosa*, if modulated by flocculosin, were dependent on other factors stimulating the growth and development of the yeast‐like fungus in the presence of powdery mildew colonies.

Recently, *P. flocculosa* was fully sequenced and annotated and its genome compared to that of closely related Ustilaginales including *U. maydis* (Lefebvre *et al*., 2013). Interestingly, on the one hand, its genome was highly similar to that of the smut fungi, with the notable exception of the loss of a few key CSEPs reported to be involved in the virulence of *U. maydis*; this led to the conclusion that this accounted for the different lifestyles between the two fungi. On the other hand, the *P. flocculosa* genome contains a few unique features such as 200 CSEPs, and lytic enzymes that could explain its singular activity against powdery mildews. However, no evidence supporting this hypothesis has been found to date.

We posit that the specific antagonistic activity of *P. flocculosa* against powdery mildews might be deciphered by studying the simultaneous transcriptomic responses of all three organisms over the development of the tritrophic interaction *P. flocculosa–B. graminis*–*H. vulgare*. We report here that CSEPs produced by *P. flocculosa* are involved in its interaction against *B. graminis*, and that *B. graminis* responds by increasing quickly and transiently its virulence against the host, until it collapses. We conclude that the BCA exploits *B. graminis* as a conduit to the plant in a hitherto unsuspected strategy, we define here as hyperbiotrophy.

## Materials and Methods

### *Pseudozyma flocculosa* and powdery mildew‐infected plant material

*Pseudozyma flocculosa* (Traquair, Shaw and Jarvis) Boekhout and Traquair (DAOM 196992) was grown in flasks in YMPD medium (yeast extract, malt extract, peptone, and dextrose; yeast extract 3 g l^−1^, malt extract 3 g l^−1^, peptone water 5 g l^−1^, and dextrose 10 g l^−1^) following Hammami *et al*. (2008). The culture was harvested at four different times spanning different phases corresponding to germination (2 h), exponential growth (8 h), flocculosin production under nutrient depletion (18 h) and maturation of sporidia (30 h). Samples collected *in vitro* were used for comparison with *P. flocculosa* transcriptome dynamics during the tripartite bioassay.

Barley (*Hordeum vulgare* L. cv Foster) plants displaying high susceptibility to *B. graminis* were used for this study. Barley seedlings were grown in a glasshouse under semi‐controlled conditions (18 h 24°C : 6 h 18°C, day : night, light : dark cycle). Three‐week‐old seedlings were exposed to natural infection with *B. graminis*. The fungus was allowed to grow on the surface of leaves under moist conditions for 4–5 d until it covered *c*. 50% of the leaf surface.

### Inoculation with *P. flocculosa* and sampling strategy

Diseased leaves were inoculated by spraying uniformly until runoff a suspension of *P. flocculosa* sporidia (*c*. 2 × 10^7^ cells ml^−1^) obtained from a 3‐d‐old culture grown in YMPD broth. The solution was left to evaporate for 20 min and the plants were covered with plastic bags to maintain a relative humidity over 90%. Plants were sampled at 0, 2, 4, 8, 12, 24 and 36 h post‐inoculation (hpi) with *P. flocculosa*.

### RNA extraction, libraries construction and sequencing

For the transcriptome analyses, leaves of four individual plant replicates of each treatment were collected, immediately frozen in liquid nitrogen and stored at −80°C.

Total RNA was extracted using Trizol™ reagent (Invitrogen) and RNeasy^®^ Mini purification kit (Qiagen) including DNAse treatment as per manufacturer's instructions. Before labelling, RNA quality and concentration were checked by agarose gel electrophoresis, spectrophotometry (Nanodrop ND‐1000; NanoDrop Technologies, Wilmington, DE, USA) and ultimately, by an Agilent 2100 Bioanalyzer™ (Agilent Technologies, Palo Alto, CA, USA).

RNA‐seq libraries were generated using the TruSeq^®^ RNA‐Seq Sample Preparation kit according to the manufacturer's protocol (Illumina Inc., San Diego, CA, USA). This kit included a polyA mRNA purification step. After quality control of the indexed cDNA libraries, multiplexed sequencing was carried out on an Illumina HiSeq™ 2000 platform by Genome Quebec (McGill University, Montréal, Canada). All of the primary sequencing data can be found in the DDBJ Sequence Read Archive (DRA) under the accession no. PSUB006820.

### RNA‐Seq data analysis

#### Raw reads processing and high‐quality reads alignment to the reference genomes

Poly‐A, adaptor sequence contaminants and low‐quality bases (Q < 15) were trimmed from Illumina reads in FASTQ format using the RNA‐seq analysis tool of CLC Genomics Workbench v8 (CLC Bio, Aarhus, Denmark) before further processing. All cleaned reads > 40 bp in length were aligned to the *P. flocculosa* reference genome; the remaining unmapped reads were then aligned to *B. graminis* and *H. vulgare* reference genomes in that order. The following criteria were used to map the unique sequence reads: mismatch cost of 2; insert cost of 3; deletion cost of 3; minimum length fraction of 0.9 and minimum similarity fraction of 0.8. Raw read counts and mapping distribution for each condition are given in Supporting Information Table S1.

#### Gene expression, differential gene expression analyses and clustering

In order to estimate the expression levels, data were expressed as reads per kilobase per million mapped reads (RPKM) by calculating the mapped reads (total exon reads/total mapped reads in millions × exon length in kb) for each gene. After normalization, data were log_2_‐transformed (0.25 was added to each RPKM number to deal with zero counts) for differential expression analysis. Significant differences in gene expression were detected using the EdgeR algorithm implemented in the CLC Genomics Workbench that utilizes the Exact Test developed by Robinson & Smyth (2008). The *P*‐value threshold was determined by the false discovery rate (FDR) to account for multiple tests of significance. A FDR threshold ≤ 0.01 was adopted to judge the significance of the gene expression change throughout the tripartite bioassay. In order to identify general trends during the tripartite bioassay, a hierarchical clustering of features was also generated for each organism using normalized expression values (mean expression level was subtracted). Clusters with similar expression patterns based on Manhattan distances were identified. Given that we had more than three replicates for each treatment, quantitative PCR validation was deemed unnecessary (Fang & Cui, 2011).

#### Functional annotation and enrichment analyses

The web‐based AgriGO tool (Du *et al*., 2010) was used to obtain Gene Ontology (GO) annotations and to perform singular enrichment analyses (FDR *P*‐value ≤ 0.05) of genes of *P. flocculosa*,* B. graminis* and *H. vulgare* differentially expressed during the tripartite interaction.

### Photosynthesis measurements

In order to validate some of the transcriptomic responses observed with the plant, photosynthesis measurements were conducted on the uppermost, fully expanded infected leaf of at least five plants at 0, 12, 24 and 36 hpi with *P. flocculosa*, as well as on five noninfected plants. All measurements were made between 9 h00 and 11 h00 am with a Li‐Cor 6400 portable system (Li‐Cor Inc., Lincoln, NE, USA). The internal LED light source was set at 500 μmol m^−2^ s^−1^ to ensure a constant uniform light across samples and leaf temperature was maintained at 25°C. In the cuvette, CO_2_ concentration was 383 ± 2 μmol mol^−1^ and relative humidity at 60% ± 1. Once steady‐state gas exchange rates were observed, net photosynthetic rate was recorded and thereafter normalized by the actual leaf surface area enclosed in the chamber.

### Light and transmission electron microscopy observations

At 0, 12, 24, 36 and 48 hpi with *P. flocculosa*, 1‐cm^2^ leaf pieces were directly imaged with an Olympus SZ61 stereomicroscope (Olympus Tokyo, Tokyo, Japan) or collected for later observations. Controls consisted of infected leaves sprayed with water and kept under plastic bags.

For light microscopy and transmission electron microscopy (TEM) observations, segments of randomly selected plants of each treatment were fixed overnight in 2.5% glutaraldehyde, 4% formaldehyde in 100 mM sodium cacosylate buffer (pH 7.3) at 4°C and postfixed for 1 h with 1% osmium tetroxide before dehydration in a grade ethanol series and embedding in LR‐white resin.

Sections were cut at 1 μm thick, stained with toluidine blue and finally imaged with a ZEISS Axioscope microscope (Zeiss, Din Mills, Ontarion, Canada). For TEM imaging, ultrathin sections (100 nm) collected on nickel gris were stained with uranyl acetate and observed with a Jeol 1200 EX microscope (Jeol, Tokyo, Japan).

For scanning electron microscopy, leaf segments were immersed in 100% dry methanol for 10 min, followed by 2 × 30 min changes in 100% dry ethanol. Ethanol was then used for critical point drying of the tissue. Finally, samples were mounted on aluminium stubs, coated with *c*. 20 nm of gold‐palladium and imaged with a Jeol JSM6360LV Scanning Electron Microscope (Jeol) at 15 kV accelerating voltage.

## Results

### Dynamic changes in phenotypes of the *P. flocculosa–B. graminis*–*H. vulgare* interaction

In order to examine relevant and specific data, sampling of the tritrophic interaction *P. flocculosa*–*B. graminis*–*H. vulgare* was optimized to highlight both phenotypic and transcriptomic responses of all three interacting organisms. Preliminary experiments indicated that no distinct or uniform pattern emerged within the first 10 h following inoculation of *P. flocculosa* (results not shown).

**Figure 1:**
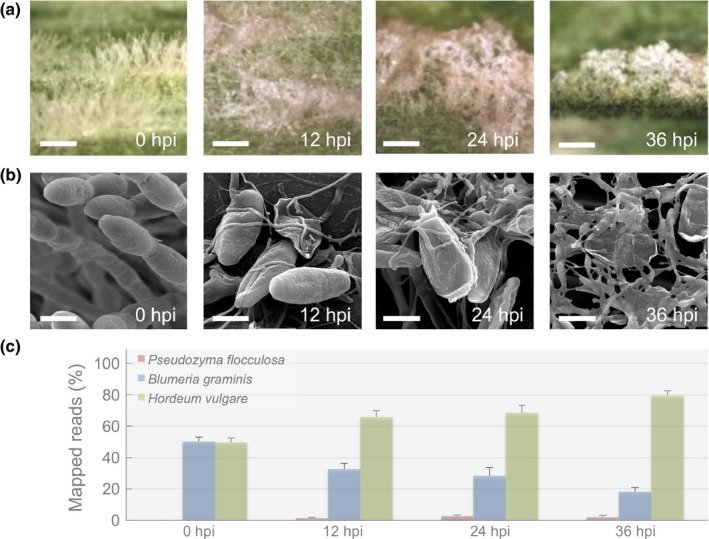
Rapid changes in phenotypes during the tripartite interaction *Pseudozyma flocculosa*–*Blumeria graminis* f.sp. *hordei*–*Hordeum vulgare*. (a) Optical stereomicroscopic observations of the antagonism of *P. flocculosa* on *B. graminis* colonies over time (bar, 400 μm). (b) Scanning electron microscopy observations of *B. graminis* colonies progressively invaded by *P. flocculosa* (bar, 10 μm). (c) Percentage of mapped reads for all three interacting organisms over different time points following *P. flocculosa* inoculation on *B. graminis* colonies. Each value is the mean ± SE (*n *≥* *3).

At 12 hpi, an initial collapse of *B. graminis* conidial chains was observed, followed by the agglutination of conidia and conidiophores at 24 hpi, which coincided with a faint discoloration on the surface of the colonies, typical of *P. flocculosa* presence (Fig. 1a). By 36 hpi, *B. graminis* colonies were completely overgrown by *P. flocculosa* (Fig. 1a). At higher magnification under SEM, mycelial filaments of *P. flocculosa* were clearly visible throughout the *B. graminis* colonies at 12 hpi, a clear indication of its nascent developmental stage following germination (Fig. 1b); by 24 hpi, *P. flocculosa* mycelium had extensively colonized *B. graminis* structures and, as expected, no evidence of penetration or coiling could be observed; finally, after 36 h, the *P. flocculosa* had completely overtaken *B. graminis* and initiated the production of sporidia (Fig. 1b).

In terms of raw transcriptomic data, an equal proportion of reads mapped to either *B. graminis* or *H. vulgare* reference genomes at 0 hpi (Fig. 1c). After inoculation with *P. flocculosa*, the proportion of reads that mapped onto *B. graminis* genome dropped to about one third of the total mapped reads (Fig. 1c). For *P. flocculosa*, reads were first detectable at 12 hpi and more than doubled at 24 hpi (1.3–2.9% total) (Fig. 1c), a time where the BCA appeared to be the most active (Fig. 1b). Interestingly, both reads from *B. graminis* and *P. flocculosa* decreased noticeably at 36 hpi (Fig. 1c) coinciding with the suppression of *B. graminis* (Fig. 1a,b).

### Transcriptome profiling of *P. flocculosa*

We exploited the extensive body of work on several aspects of *P. flocculosa* (Hammami *et al*., 2008; Lefebvre *et al*., 2013) to devise a strategy for the analysis and interpretation of transcriptome dynamics. In this regard, our analyses were greatly facilitated by the previous identification of several genes, or gene groups that were proposed to be relevant to its specific lifestyle.

Of the 6877 genes annotated in the *P. flocculosa* genome (Lefebvre *et al*., 2013), 91 ± 2% were expressed *in vitro* ≥ 1 RPKM in at least one of the four sampling times, and 94 ± 1% in biocontrol conditions (see Table S2 for detailed expression data). A total of 3207 differentially expressed genes (DEG) were identified (fold‐change ≥ ∣2∣ between two sampling times; FDR *P*‐value ≤ 0.01) in *P. flocculosa* during the course of the tripartite bioassay. In addition, many of the highly expressed genes by sporidia at 30 h *in vitro*, were also observed *in planta* at 36 hpi when *P. flocculosa* started producing sporidia (Table S2; Fig. 1b).

#### *Pseudozyma flocculosa* mode of action is unrelated to plant pathogen relatives

As many as 285 genes showed no expression *in planta*; these included both *b* locus‐encoded homeodomain proteins, pf03264 and pf03265, orthologs of genes involved in mating and subsequent pathogenicity in *U. maydis*. In addition, none of the 47 putative pathogenesis associated genes associated to smut fungi and found in *P. flocculosa* genome exhibited higher expression values *in planta* than *in vitro* (Table S3).

#### Flocculosin appears to have limited role in biocontrol activity

**Figure 2:**
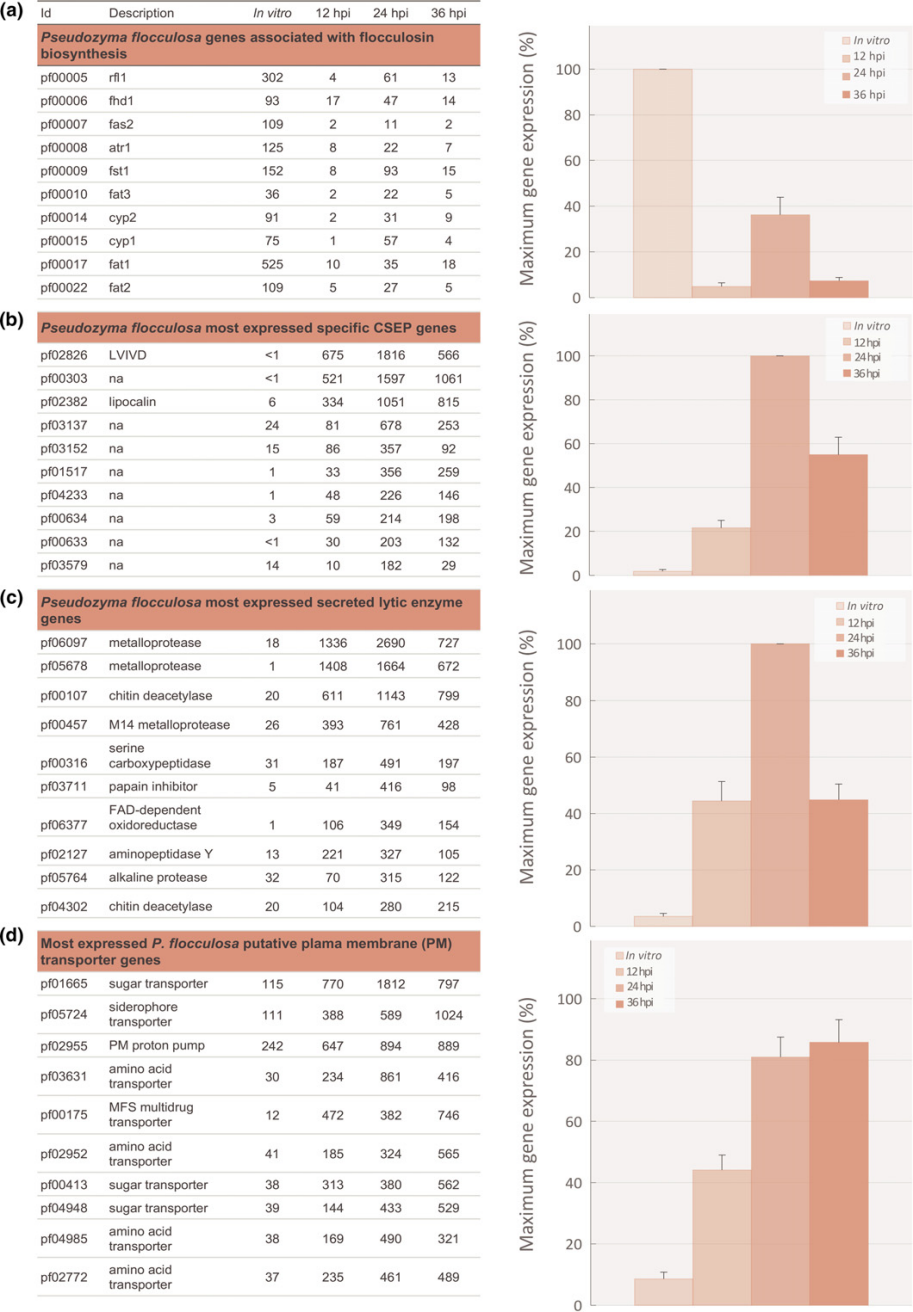
Transcriptome analysis of *Pseudozyma flocculosa* during the tripartite interaction *P. flocculosa*–*Blumeria graminis* f.sp. *hordei*–*Hordeum vulgare*. Transcriptome analysis *P. flocculosa* during the tripartitre interaction *P. flocculosa*–*B. graminis*–*H. vulgare* revealed: (a) the limited transcription of genes associated with flocculosin biosynthesis in *P. flocculosa* during the tripartite interaction; (b) the rapid induction of genes encoding candidate secreted effector proteins (CSEPs) specific to the biocontrol agent; (c) the rapid induction of genes encoding putative secreted lytic enzymes and (d) the rapid induction of genes encoding putative transmembrane transporters. Average reads per kilobase per million mapped reads (RPKM) data of genes of the flocculosin cluster and of genes significantly differentially expressed (FDR *P*‐value ≤ 0.01) during the tripartite interaction *P. flocculosa*–*B. graminis*–*H. vulgare* at 12, 24 and 36 h post‐inoculation (hpi) on *B. graminis* colonies compared to *in vitro* cultures (*n *≥* *3) are given in the table on the left. On the right, histograms showing the average relative (%) cluster expression at each time point based on the highest level of expression for each gene as a measure to showcase the trend in expression dynamics. Each value is the mean ± SE (*n *=* *10 genes).

We analyzed the expression of all 11 genes involved in flocculosin synthesis, the antifungal molecule long associated with the biocontrol arsenal of *P. flocculosa*. All genes were weakly expressed compared to *in vitro* conditions in the early stage of interaction at 12 hpi (Fig 2a, S1). A generalized increase in expression was observed at 24 hpi, albeit at much lower levels than *in vitro* before receding to lower levels at 36 hpi.

#### Unique *P. flocculosa* CSEPs and other secreted proteins are involved in antagonism against *B. graminis*

Among the highest DEGs observed, many belonged to CSEPs reported to be unique to *P. flocculosa* (Lefebvre *et al*., 2013). From those CSEPs, we identified a group of 10 highly expressed genes whose analysis confirmed a similar pattern of expression (Figs 2b, S2). In fact, those effectors were highly expressed during contact with *B. graminis*, contrasting with a complete or near complete lack of expression *in vitro*. Expression increased over time to reach a peak at 24 hpi and subsided at 36 hpi when *P. flocculosa* had completely overtaken *B. graminis* (see Fig. 1). Of particular importance, three transcripts, pf02826, pf00303 and pf02382, had a differential fold‐change > 175 when compared to *in vitro* conditions.

Within the secretome, several lytic enzyme genes also were found to be strongly induced in presence of *B. graminis* (Figs 2c, S3). As with CSEPs above, their expression was consistently higher at 24 hpi. In particular, two metalloproteases, pf06097 and pf05678, showed the highest RPKM values of the group.

#### Importance of *P. flocculosa* transporters revealed in the interaction with *B. graminis*

Considering that transporter proteins play an important role in substrate acquisition, a particular emphasis was placed on their analysis. This revealed that several genes coding for sugar and amino acid transporters were upregulated during the rapid growth phase of the BCA compared to growth *in vitro* (Figs 2d, S4).

### Transcriptome profiling of *B. graminis*

The transcriptomic response of *B. graminis* to *P. flocculosa* presence was broad: 1872 genes exhibited differential expression levels (Table S4; fold‐change ≥ ∣2∣ between two sampling times, FDR *P*‐value ≤ 0.01) throughout the sampling period including 1082 that were upregulated.

#### Generalized increase of transport system in *B. graminis* appears to benefit *P. flocculosa*

**Figure 3:**
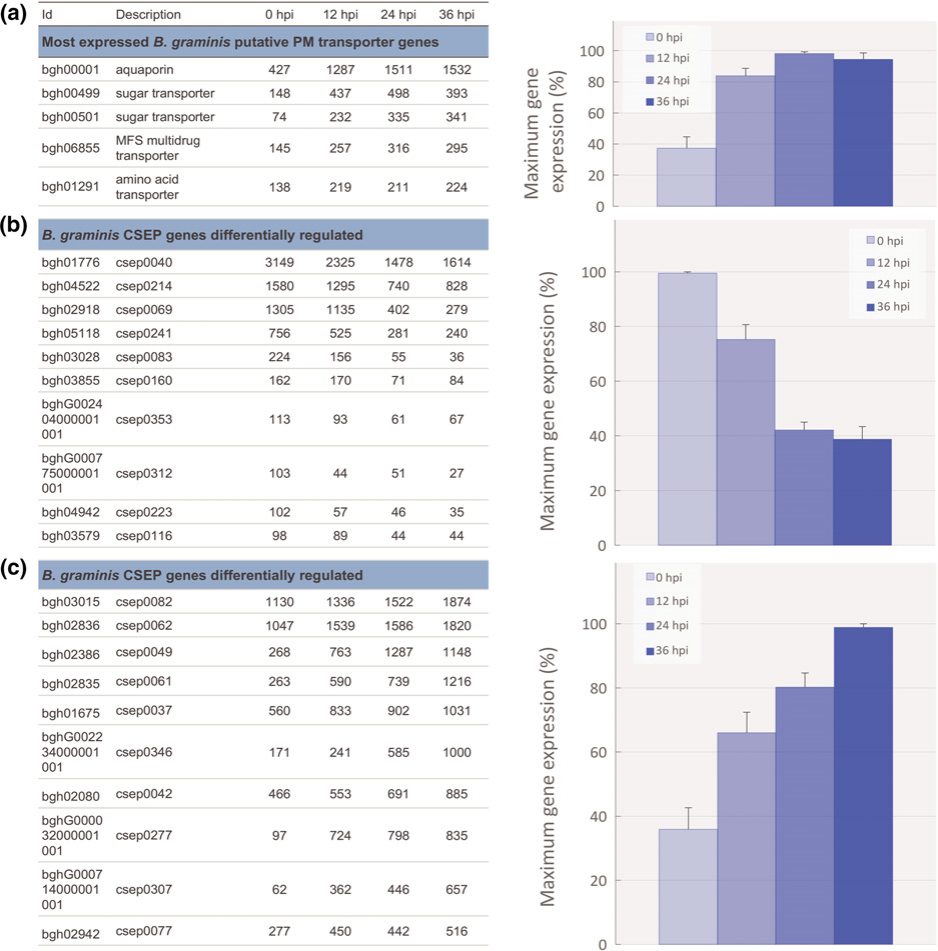
Transcriptome analysis of *Blumeria graminis* f.sp. hordei during the tripartite interaction *Pseudozyma flocculosa*–*B*. *graminis*–*Hordeum vulgare*. Transcriptome analysis *B. graminis* during the tripartitre interaction *P. flocculosa*–*B. graminis*–*H. vulgare* revealed: (a) the increasing transcription pattern of genes encoding putative transmembrane transporters; (b) the decreasing expression pattern of conidia‐ and hyphae‐specific candidate secreted effector protein (CSEP) genes and (c) the increasing expression pattern of haustoria‐specific CSEP genes. Average reads per kilobase per million mapped reads (RPKM) data of genes significantly differentially expressed (FDR *P*‐value ≤ 0.01) during the tripartite interaction *P. flocculosa*–*B. graminis*–*H. vulgare* at 0, 12, 24 and 36 h post‐inoculation (hpi) on *B. graminis* colonies (*n *≥* *3) are given in the table on the left. On the right, histogram showing the average relative (%) cluster expression at each time point based on the highest level of expression for each gene as a measure to showcase the trend in expression dynamics. Each value is the mean ± SE (*n *= number of genes presented in the table on the left).

AgriGO singular enrichment analysis of the differentially expressed genes revealed the overrepresentation of GO terms: ‘transmembrane transporter function’, ‘membrane component’ and ‘carbohydrate metabolic process’ among the genes induced in presence of *P. flocculosa* (Fig. S5). These results were seemingly in contradiction with the phenotypes observed. Genes associated with *B. graminis* nutrient acquisition system were over‐stimulated in spite of a vegetative development obviously altered by the rapid growth of *P. flocculosa* (Figs 3a, S6). In fact, several sugar and amino acid transporters in *B. graminis* were found to be upregulated in synchrony with the dynamic regulation of similar transport genes of *P. flocculosa* (see Fig. 2d).

#### Unexpected patterns of CSEP expression are observed in *B. graminis*

Several CSEPs were identified in the sequencing and annotation of *B. graminis* genome (Spanu *et al*., 2010). These were implicated in the virulence of the pathogen. For this reason, they were emphasized in our analyses. Based on cluster analysis and expression dynamics, two clear and distinct patterns emerged among the most expressed CSEPs.

In a first group of CSEPs, all reported as encoding proteins produced by conidia and hyphae (Pedersen *et al*., 2012), we observed a declining level of expression in *B. graminis* colonies following *P. flocculosa* inoculation, a pattern well‐aligned with the early degradation of *B. graminis* mycelium and conidia (Figs 3b, S7). In addition, another 15 CSEPs were similarly repressed in the presence of *P. flocculosa* (FDR *P*‐value ≤ 0.01; Table S4).

The second group had arguably the most counterintuitive pattern. Based on cluster analysis, 112 CSEPs were found to have an increased expression over time (FDR *P*‐value ≤ 0.01) in spite of the obvious stress imposed by the presence of *P. flocculosa*. Analysis of the ten most expressed ones revealed that they were all of haustorial origin (Bindschedler *et al*., 2009, 2011), and that they steadily increased over the base level maintaining infection with barley (Figs 3d, S8), even when ectotrophic structures were physically destroyed by *P. flocculosa* (Fig. 1).

### Profiling of *Hordeum vulgare* transcriptome and physiology

#### A tale of two pathogens?

**Figure 4:**
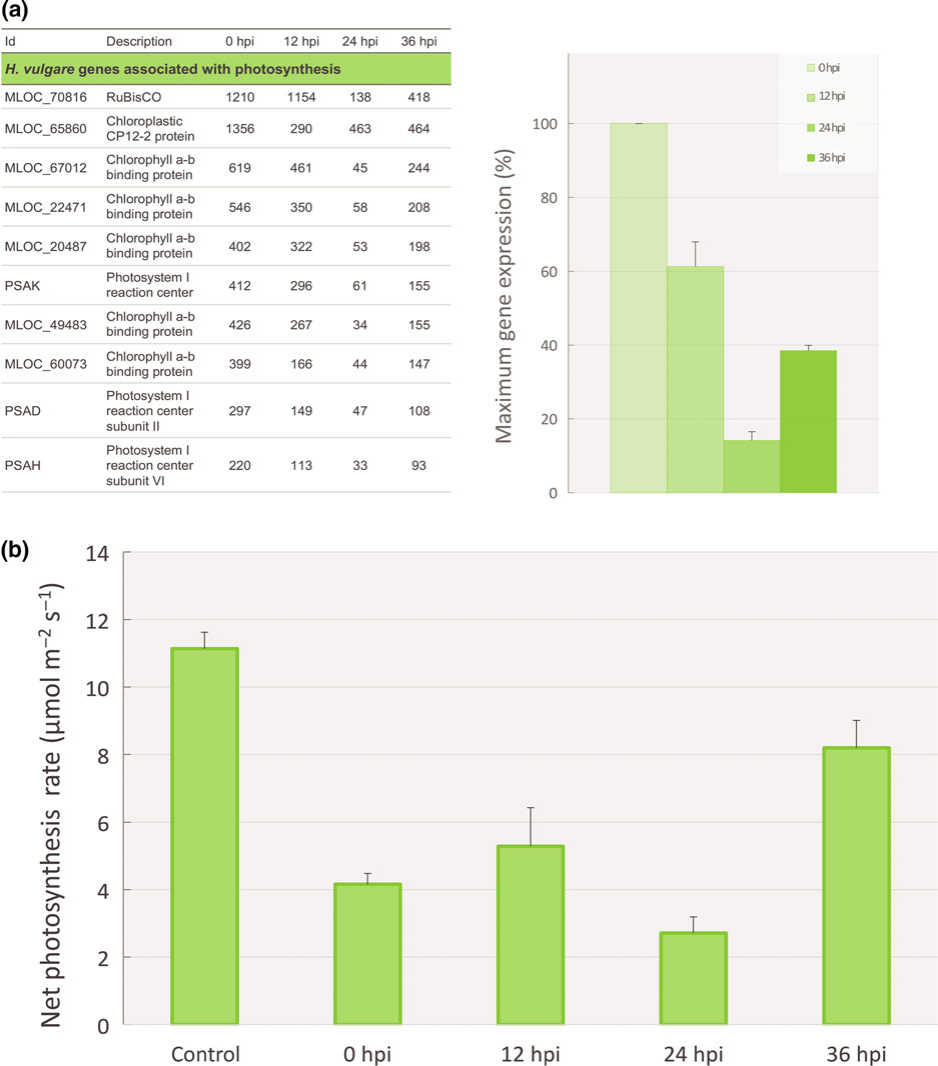
Effect of *Pseudozyma flocculosa* on the expression profile of the most expressed photosynthesis‐related genes and on net photosynthesis rate in *Hordeum vulgare* during the tripartite interaction *P. flocculosa*–*Blumeria graminis* f.sp. *hordei*–*H. vulgare*. (a) Average reads per kilobase per million mapped reads (RPKM) data of the most expressed photosynthesis‐related genes of *H. vulgare* significantly differentially expressed (FDR *P*‐value ≤ 0.01) during the tripartite interaction *P. flocculosa*–*B. graminis*–*H. vulgare* at 0, 12, 24 and 36 h post‐inoculation (hpi) on *B. graminis* colonies (*n *≥* *3) are given in the table on the left. The histogram shows the average relative (%) cluster expression at each time point based on the highest level of expression for each gene as a measure to showcase the trend in expression dynamics. Each value is the mean ± SE (*n *=* *10 genes). (b) Net photosynthesis rate (μmol m^−2^ s^−1^) of *H. vulgare* leaves during the tripartite interaction. Each value is the mean ± SE (*n *≥* *5 plants).

Photosynthesis deregulation is one of the most reliable markers of powdery mildew presence on a plant. At the transcriptomic level, ‘Photosynthesis’, ‘secondary metabolic process’ and ‘response to stimulus’ were the most significantly over‐represented GO terms (Fig. S9) among the 1259 *H. vulgare* DEGs observed in response to *P. flocculosa* inoculation (Table S5; fold‐change ≥ ∣2∣ between two sampling times, FDR *P*‐value ≤ 0.01). Among other common and important DEGs in barley, many defense‐related proteins were observed within highly expressed genes (Fig. S10; Table S5). Their expression increased over time consistent with the phenotype of a plant fighting off infection throughout the experimental period. Most importantly, the pattern of expression of photosynthesis‐related genes showed a sharp reduction over time that culminated at 24 hpi before going back up at 36 hpi (Figs 4a, S11).

#### Photosynthesis measurements

Based on barley transcriptomic responses, photosynthetic rates were measured on plants subjected to the same treatment to determine if the fungal interactions influenced the plants reaction. A pattern of declining photosynthetic rate of leaves was observed up to 24 hpi, followed by a resurgence in the following 12 h (36 hpi; Fig. 4b), in line with the reduced activity of both *B. graminis* and *P. flocculosa*.

### Light microscopy and TEM observations of the interaction

**Figure 5:**
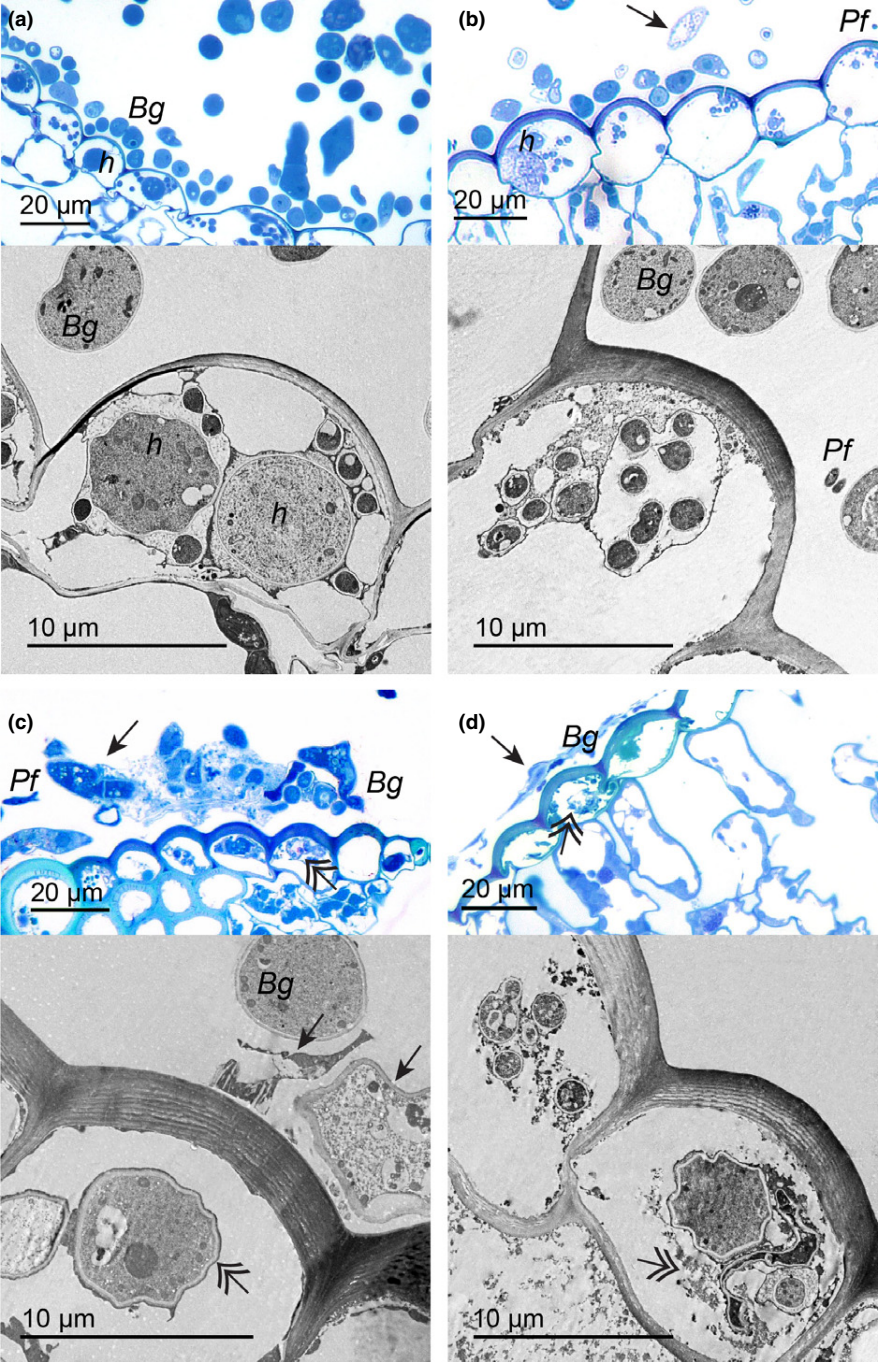
Light and transmission electron microscopy (TEM) of the interaction *Pseudozyma flocculosa–Blumeria graminis* f.sp. *hordei*–*Hordeum vulgare* reveal phenotypic responses supporting transcriptomic results. (a) Light and TEM. Healthy surface hyphae and conidia (*Bg*), and haustoria (*h*) of *B. graminis* are visible in barley cells before inoculation with *P. flocculosa* (0 hpi). (b) At 12 hpi, presence of *P. flocculosa* (*Pf*) as well as *B. graminis* empty hyphae are observed on the surface (arrows; light), whereas haustoria and digitations are seemingly healthy (TEM). (c) At 24 hpi, surface hyphae and conidia are clearly degraded (arrow; light), in barley cells, the haustoria also show signs of degradation (double arrow; TEM) along with a thicker extra‐haustorial matrix. (d) At 36 hpi, a denser layer of dead hyphae is evident on the leaf surface (arrow; light), and haustorial structures are convoluted when not completely plasmolyzed (double arrow; light and TEM).

Light and transmission electron microscopy observations throughout the tripartite interaction yielded phenotypes that supported the transcriptomes of the three organisms (Fig. 5). Compared with the control (Fig. 5a), haustoria in barley cells presented digitations seemingly unaltered whereas a few ectotrophic hyphae were devoid of cytoplasm, 12 h after *P*. *flocculosa* inoculation (Fig. 5b). At 24 hpi, surface conidia and hyphae had clear signs of degradation, whereas haustorial bodies started to show evidence of alteration along with a thicker extra‐haustorial matrix (Fig. 5c). At 36 hpi, a clear layer of dead hyphae was observed on the leaf surface and, when still integral, haustorial structures were convoluted and sometimes collapsed (Fig. 5d).

## Discussion

Historically, the interaction between a plant pathogen and a biocontrol agent (BCA) has been described as one of competition, antibiosis and/or parasitism based on 1D or 2D analyses (Paulitz & Bélanger, 2001). We present here the first comprehensive transcriptomic responses of a tritrophic interaction, BCA–powdery mildew–plant, highlighting unique and unsuspected interdependent reactions at each trophic level.

Since its discovery, *Pseudozyma flocculosa* has always been described as a superior antagonist because of its faster mode of action than other reported BCAs of powdery mildews such as *Ampelomyces quisqualis* or *Tilletiopsis* spp. (Hajlaoui & Bélanger, 1993; Dik *et al*., 1998). In our experiments, we reproduced the expected phenotypic responses where powdery mildew structures were quickly colonized and killed within a period of 24 h. For its part, *P. flocculosa* development was visible within 12 h post‐inoculation (hpi), peaked at 24 hpi and started receding at 36 hpi when powdery mildew colonies were seemingly saturated. This pattern is consistent with previous reports where longer bioassays have further shown that *P. flocculosa* populations are reduced to epiphytic levels once powdery mildew colonies are dead or absent (Neveu *et al*., 2007; Clément‐Mathieu *et al*., 2008; Hammami *et al*., 2011). Interestingly, the transcript proportions of both *P. flocculosa* and *Blumeria graminis* matched the observed phenotypes over time.

Comparative genomics has recently highlighted the remarkable similarity between *P. flocculosa* and smut fungi belonging to the Ustilaginomycetes (Lefebvre *et al*., 2013) but could only speculate about the factors that brought about such divergent lifestyles. In *Ustilago maydis*, the mating factor bE/bW heterodimer acts as the central regulator for pathogenicity (Romeis *et al*., 2000). In the course of our bioassays, the two *P. flocculosa* bE/bW orthologs were never transcribed, nor were numerous downstream genes or the putative pheromone precursor and receptors, which are determinant for the sexual reproduction and the development of the dikaryotic pathogenic state in plant pathogens. Together with the loss of key effectors for pathogenicity (Lefebvre *et al*., 2013), these results confirm that *P. flocculosa* has indeed lost the ability to be a plant pathogen in the same category as smut fungi.

Ever since its discovery, *P. flocculosa* has been described as a biocontrol agent of powdery mildews acting through antibiosis, the latter mode of action being entirely related to its ability to produce an unusual glycolipid through the activation of a conserved cluster of genes also found in the *U. maydis* genome (Teichmann *et al*., 2011a; Bélanger *et al*., 2012). Despite its potent antifungal properties (Mimee *et al*., 2005), the role of this glycolipid, flocculosin, in the biocontrol activity of *P. flocculosa* was recently questioned because its synthesis did not appear to be synchronized with *P. flocculosa* development on powdery mildews (Marchand *et al*., 2009; Hammami *et al*., 2011). In the present study, the 11 genes regulating flocculosin synthesis were strongly expressed *in vitro* but to a much lesser extent during the tripartite bioassay, especially at 12 hpi at a time where one would expect an early release facilitating access to powdery mildew colonies. Therefore, transcriptomic data support the notion that flocculosin is not a key factor in the biocontrol activity of *P. flocculosa*, namely in the context of its specificity to powdery mildews. Given its link to ustilagic acid produced by *U. maydis* and other related Ustilaginales (Kulakovskaya *et al*., 2005; Teichmann *et al*., 2007), conservation of the trait among those fungi appears more likely related to protection of an ecological niche on the leaf surface during the epiphytic stage.

Beyond the common genomic features, Lefebvre *et al*. (2013) reported several unique genes in *P. flocculosa*, including > 200 candidate secreted effector proteins (CSEPs) that we targeted to start analyzing transcripts of potential interest. The notion that some of them could be important for the antagonistic activity of *P. flocculosa* raised interesting possibilities because this phenomenon, although widely described in plant–pathogen interactions, has never been reported in fungal–fungal interactions. It was therefore fascinating to observe that several *P. flocculosa* CSEPs were among the most transcribed genes during the tripartite interaction. Their pattern of expression also was consistent with the biocontrol activity of *P. flocculosa*, thereby supporting their role as potential virulence factors against *B. graminis*. As a matter of fact, the repertoire of species‐specific candidate effectors in *P. flocculosa* is nearly three times as great compared to its smut relatives (*U. maydis*,* U. hordei* or *Sporisorium reilianum*; Lefebvre *et al*., 2013), thus suggesting different functions. Indeed, on the one hand, no orthologs of *U. maydis* effectors, actually characterized for their essential role in plant pathogenicity, were expressed in *P. flocculosa*. These include Pep1 (pf02348), Cmu1 (pf04511) or the Pit cluster (pf04022, pf04023) (Djamei *et al*., 2011; Doehlemann *et al*., 2011). On the other hand, several CSEPs, namely pf02826, pf00303 and pf02382, were highly and exclusively expressed during contact with powdery mildew structures, a result providing strong evidence that this fungal–fungal interaction is governed by effector proteins. For instance, the presence of a lipocalin PFAM domain on pf02382 is of particular interest. Lipocalins are secreted proteins that bind and transport small molecules, which suggests that pf02382 may be involved in the dissemination, sequestration of various compounds or also the capture for its own benefit of molecules such as iron‐charged siderophores (Fischbach *et al*., 2006).

Our results also revealed that the expression of many transcripts coding for lytic enzymes was specifically elicited following contact with *B. graminis*. On the one hand, although lytic enzymes have often been associated with direct parasitism in some BCAs (Whipps, 2001), this expression is unlikely to be related to this mode of action because the cycle of *P. flocculosa* is too short and no penetration of *P. flocculosa* inside powdery mildew structures has ever been observed (Hajlaoui & Bélanger, 1993; Bélanger *et al*., 2012). On the other hand, pf00107 and pf04302 proteins could potentially play a major role in rapid cell wall permeation as suggested for a chitinase of *Trichoderma harzianum* (Zeilinger *et al*., 1999). In addition, similar to the ones observed here, a secreted metallopeptidase with antifungal activity has been recently characterized as an essential component of *T. guizhouense*'s ability to parasite the causative agent of banana wild disease (Zhang *et al*., 2016). Metalloproteases such as pf06097, pf05678 and pf00457 are also reported to be involved in the breakdown of nutrient sources from host cells (Druzhinina *et al*., 2012).

The previous observations are consequent with a pattern of nutrient acquisition and accordingly, major changes in the transmembrane transport system of *P. flocculosa* were noted. For instance, contact with *B. graminis* clearly triggered an accumulation of sugar and amino‐acid transporter transcripts in a manner reminiscent of plant surface signals inducing *U. maydis* transporters to provide the pathogen with resources diverted from the plant metabolism (Lanver *et al*., 2014). Interestingly, the most expressed sugar transporter gene, pf01665, is a close homolog of the *U*. *maydis* Hxt1 transporter required for virulence (Schuler *et al*., 2015). In the rust fungus *Uromyces fabae*, HXT1 is a haustorium‐specific protein involved in sugar efflux from the infected plant tissue to the pathogen (Voegele *et al*., 2001). The expression profile of pf03631, a Gap1 general amino‐acid permease homolog, mirrors Hxt1 in this study. In *Saccharomyces cerevisiae*, GAP1 is the major transporter of amino acid for use as a nitrogen source (Magasanik & Kaiser, 2002; Donaton *et al*., 2003). Considering their expression levels at 24 hpi, pf01665 and pf03631 are likely to have similar functions in *P. flocculosa*, whereby these specific permeases with putative sensor functions may help to assimilate nutrients directly from *B. graminis*.

When looking into the *B. graminis* transcriptomic profile following inoculation with *P. flocculosa*, several responses were seemingly incongruent with the observed interaction phenotypes. Although *B. graminis* was clearly being overwhelmed by *P. flocculosa* and its external structures plasmolysed, several transmembrane transporters like the haustorial‐specific sugar transporter bgh00499, known to be involved in pathogenesis (Bindschedler *et al*., 2011; Hacquard *et al*., 2013), exhibited an increase in expression as early as 12 hpi to a level that was maintained throughout the interaction. Considering the rapid development of *P. flocculosa* over that time period and a concomitant increase in transporters activity in both fungi, these results could suggest a diversion of nutrients extracted by *B. graminis* from barley for the benefit of *P. flocculosa*.

The expression dynamics of *B. graminis* CSEPs was also very informative with respect to their functionality. In essence, two distinct patterns of expression were observed and converged toward different roles. In a first group, there was a regular downward trend over time following *P. flocculosa* inoculation. This pattern is at first predictable because a reduction in expression should be synchronized with *B. graminis* being antagonized. Among the CSEP genes found in this group, two of them, csep0040 and csep0214 (Pedersen *et al*., 2012), have been associated with conidia, whereas the other ones encode secreted proteins of the mycelium. By contrast, several CSEPs were found to have an increasing pattern of expression over time. Interestingly, most of these CSEPs, including the highly expressed csep0082 and csep0062, are haustoria specific (Bindschedler *et al*., 2009, 2011) and contribute to the virulence of *B. graminis*. There is a counterintuitive quality to this because it is likely that this would affect the delicate balance inherent to biotrophy established between *B. graminis* and barley cells. A possible explanation is that *P. flocculosa* exploits the *B. graminis* pathogenic machinery and exacerbates its virulence, albeit for a short period of time, for its own benefit.

The transcriptome profiling of barley plantss also revealed patterns of gene expression that suggested the interaction between *P. flocculosa* and *B. graminis* was in fact greatly influenced by the plant. For instance, it is well known that powdery mildew infection results in reduction of plant photosynthesis (Higgins *et al*., 1985; Scholes *et al*., 1994; Chain *et al*., 2009; Chandran *et al*., 2010). As such, one would expect that *P. flocculosa* antagonism of *B. graminis* would quickly alleviate the stress exerted by the pathogen onto barley leaves. However, physiological data and analysis of genes related to photosynthesis revealed a pattern that suggested otherwise. Indeed, we observed a sharp downregulation of photosynthetic genes concomitant with *P. flocculosa* rapid development within the first 24 h, followed by an upregulation at 36 hpi when the activity of *P. flocculosa* receded. This would indicate, in line with earlier observations, that *P. flocculosa* actually exploits *B. graminis* as a conduit to extract nutrients from the plant.

**Figure 6:**
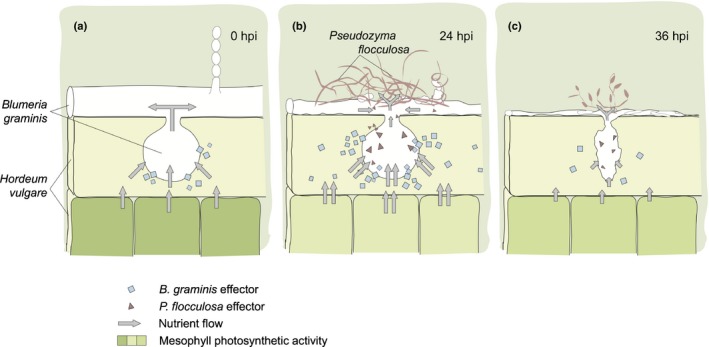
Proposed model of hyperbiotrophy in the tritrophic interaction *Pseudozyma flocculosa*–*Blumeria graminis* f.sp. *hordei*–*Hordeum vulgare*. (a) A biotrophic interaction is established between *B. graminis* and *H. vulgare* through the formation of a haustorium within the epidermal cells, and the release of effectors preventing defense reactions by the plant, and diverting nutrients from the plant to the pathogen (Chaudhari *et al*., 2014). (b) Upon contact with *B. graminis* structures, *P. flocculosa* overwhelms the pathogen colonies within 24 h, a phenomenon synchronized with the higher expression of transporters and unique *P. flocculosa* effectors, which in turn exacerbates the virulence of *B. graminis* by overstimulating its expression of effectors and the extraction of nutrients from *H. vulgare* cells to the profit of *P. flocculosa*. This hyperbiotrophic activity affects the plant transiently by further reducing gene expression linked to photosynthesis. (c) Deregulation of the delicate biotrophic balance between *B. graminis* and *H. vulgare* leads to the decline of the haustorium activity and its eventual collapse, thereby reducing the flow of nutrients toward *P. flocculosa* and halting its growth within 36 h, and at the same time allowing the plant to start recovering through increased photosynthetic activity.

Taken together, these findings have uncovered a novel mode of action within the antagonistic repertoire of biocontrol agents that we describe here as hyperbiotrophy. Indeed, against all expectations, the ultimate host target of *P. flocculosa*, appears to be the plant, a parasitism that is achieved through the powdery mildew pathogen. In Fig. 6, we propose a model that explains how the interactions between the three organisms lead to this rare form of parasitism. Upon contact with *B. graminis*,* P. flocculosa* releases an unique set of effectors, which in turn activates the virulence of *B. graminis* through an exacerbated production of haustorial effectors and deregulates its interaction with barley. This interaction accentuates the acquisition of nutrients from the plant, as shown by the increased stress on its photosynthetic ability, nutrients that are, in all likelihood, diverted toward *P. flocculosa* because the latter develops rapidly during this period whereas the ectotrophic structures of *B. graminis* become progressively destroyed. This process, as observed here and in other reports (Hammami *et al*., 2011), is very transient and lasts approximately only 24 h, its demise being linked with the collapse of *B. graminis* (see Figs 1, 5, 6). Incidentally, this model explains why *P. flocculosa* populations will be reduced to epiphytic levels in the hours following the interruption of the conduit between the plant and the pathogen (Hajlaoui *et al*., 1992; Hammami *et al*., 2011). This model is also coherent with previous observations showing that *P. flocculosa* is unable to grow on detached powdery mildew spores, or on the plant alone, and is specific to powdery mildew pathogens. The latter conclusion had puzzled scientists because flocculosin (and ustilagic acid), presumed to be the active principle of *P. flocculosa* biocontrol activity for a long time, was toxic against a wide array of fungi (Mimee *et al*., 2005; Teichmann *et al*., 2007). Future efforts should concentrate on assessing the functionality of the salient genes of *P. flocculosa* and *B. graminis*, a challenge currently hampered by the fact that neither of the fungi is amenable to transformation (Bélanger *et al*., 2012; Yan *et al*., 2012).

In conclusion, our transcriptomic analysis of the tripartite interaction *P. flocculosa*–*B. graminis*–*H. vulgare* has uncovered a complex phenomenon of hyperbiotrophy, the first such description for a biocontrol agent. Based on our observations, *P. flocculosa* would actually parasitize the plant, albeit transiently, by diverting nutrients extracted by *B. graminis* through a process involving the release of unique effectors, bringing evidence that such molecules can also influence fungal–fungal interactions.

## Author contributions

J.L. carried out physiological data measurements and transcriptomic analyses, participated in the interpretation of data and drafted the manuscript; J.L., C.L. and R.R.B. carried out optical microscopy and transmission electronic microscopic observations; G.B.R., F.L. and C.L. carried out sample collection, RNA‐Seq experiments and transcriptomic analyses; C.L. and P.D.S. were involved in interpretation of data and drafting of the manuscript; and R.R.B. conceived the study, participated in interpretation of data and writing of the article. All authors have read and approved the final manuscript.

## Supporting information

Please note: Wiley Blackwell are not responsible for the content or functionality of any Supporting Information supplied by the authors. Any queries (other than missing material) should be directed to the *New Phytologist* Central Office.

**Fig. S1 **Transcriptomic profiles of genes associated with flocculosin biosynthesis in *Pseudozyma flocculosa* during the tripartite interaction *P. flocculosa*–*Blumeria graminis* f.sp. *hordei*–*Hordeum vulgare*.
**Fig. S2 **Transcriptomic profiles of the most expressed CSEPs genes specific to *Pseudozyma flocculosa* during the tripartite interaction *P. flocculosa*–*Blumeria graminis* f.sp. *hordei*–*Hordeum vulgare*.
**Fig. S3** Transcriptomic profiles of secreted lytic enzyme genes in *Pseudozyma flocculosa* during the tripartite interaction *P. flocculosa*–*Blumeria graminis* f.sp. *hordei*–*Hordeum vulgare*.
**Fig. S4** Transcriptomic profiles of transporter genes in *Pseudozyma flocculosa* during the tripartite interaction *P. flocculosa*–*Blumeria graminis* f.sp. *hordei*–*Hordeum vulgare*.
**Fig. S5 **Gene ontology (GO) enrichment of *Blumeria graminis* differentially expressed genes during the tripartite interaction.
**Fig. S6** Transcriptomic profiles of transporter genes in *Blumeria graminis* during the tripartite interaction *Pseudozyma flocculosa*–*B. graminis* f.sp. *hordei*–*Hordeum vulgare*.
**Fig. S7** Transcriptomic profiles of conidia‐ and hyphae‐specific CSEPs in *Blumeria graminis* during the tripartite interaction *Pseudozyma flocculosa*–*B. graminis* f.sp. *hordei*–*Hordeum vulgare*.
**Fig. S8** Transcriptomic profiles of haustoria‐specific CSEPs in *Blumeria graminis* during the tripartite interaction *Pseudozyma flocculosa*‐*B. graminis* f.sp. *hordei*–*Hordeum vulgare*.
**Fig. S9 **Gene ontology (GO) enrichment of *Hordeum vulgare* differentially expressed genes during the tripartite interaction.
**Fig. S10** Transcriptomic profiles of photosynthesis‐related genes in *Hordeum vulgare*during the tripartite interaction *Pseudozyma flocculosa*–*Blumeria graminis* f.sp. *hordei*–*H. vulgare*.
**Fig. S11 **Transcriptomic profiles of the most expressed genes in *Hordeum vulgare* during the tripartite interaction *Pseudozyma flocculosa*–*Blumeria graminis* f.sp. *hordei*–*H. vulgare*.

**Table S1** Summary of RPKM values obtained from the *in vitro* experiment and the tripartite bioassay
**Table S2 **Summary of RNAseq data of *Pseudozyma flocculosa* mapped reads during the tripartite bioassay
**Table S3 **RPKM data of orthologs of *Pseudozyma flocculosa* genes related to filament formation and pathogenic development
**Table S4 **Summary of RNAseq data of *Blumeria graminis* mapped reads during the tripartite bioassay
**Table S5 **Summary of RNAseq data of *Hordeum vulgare* mapped reads during the tripartite bioassay
