# Supplementary material for: Effectors involved in fungal–fungal interaction lead to a rare phenomenon of hyperbiotrophy in the tritrophic system biocontrol agent–powdery mildew–plant
Source: New Phytol. 2017 Oct 18;217(2):713–25. doi: 10.1111/nph.14851 (PMC6079639; doi:10.1111/nph.14851)

***New Phytologist* Supporting Information**

Article title: Effectors involved in fungal-fungal interaction lead to a rare phenomenon of hyperbiotrophy in the tritrophic system biocontrol agent-powdery mildew-plant

Authors: Joan Laur, Gowsica Bojarajan Ramakrishnan, Caroline Labbé, François Lefebvre, Pietro D. Spanu and Richard R. Bélanger

Article acceptance date: 13 September 2017

The following Supporting Information is available for this article:

Fig. S1 Transcriptomic profiles of genes associated with flocculosin biosynthesis in *Pseudozyma flocculosa* during the tripartite interaction *P. flocculosa*-*Blumeria graminis* f.sp. *hordei*-*Hordeum vulgare*.

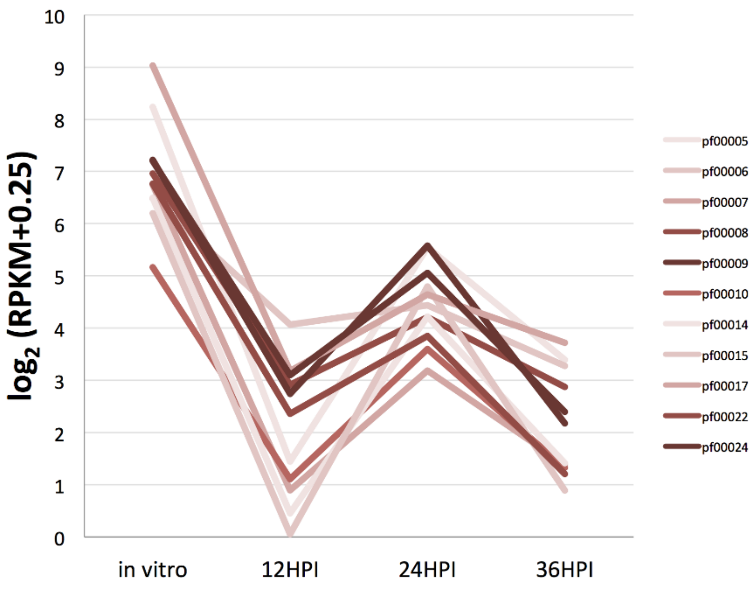

Fig. S2 Transcriptomic profiles of the most expressed CSEPs genes specific to *Pseudozyma flocculosa* during the tripartite interaction *P. flocculosa*-*Blumeria graminis* f.sp. *hordei*-*Hordeum vulgare*.

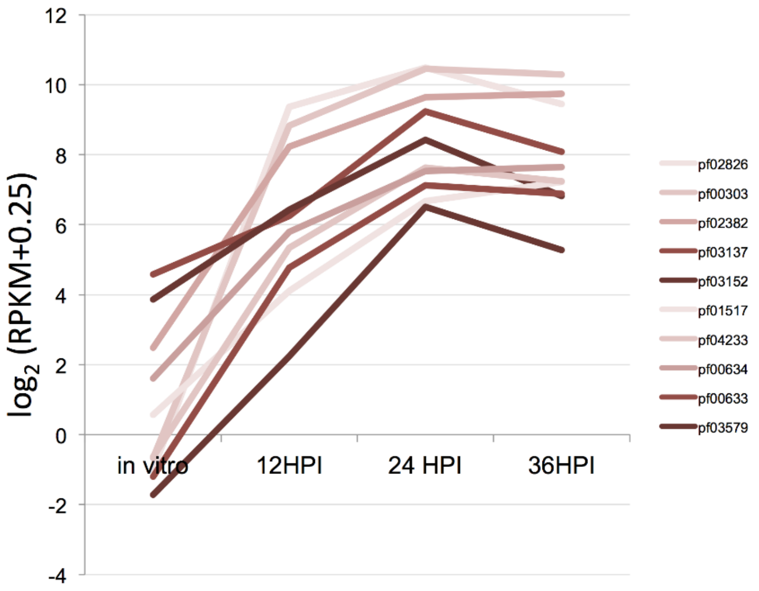

Fig. S3 Transcriptomic profiles of secreted lytic enzyme genes in *Pseudozyma flocculosa* during the tripartite interaction *P. flocculosa-Blumeria graminis* f.sp. *hordei-Hordeum vulgare.*

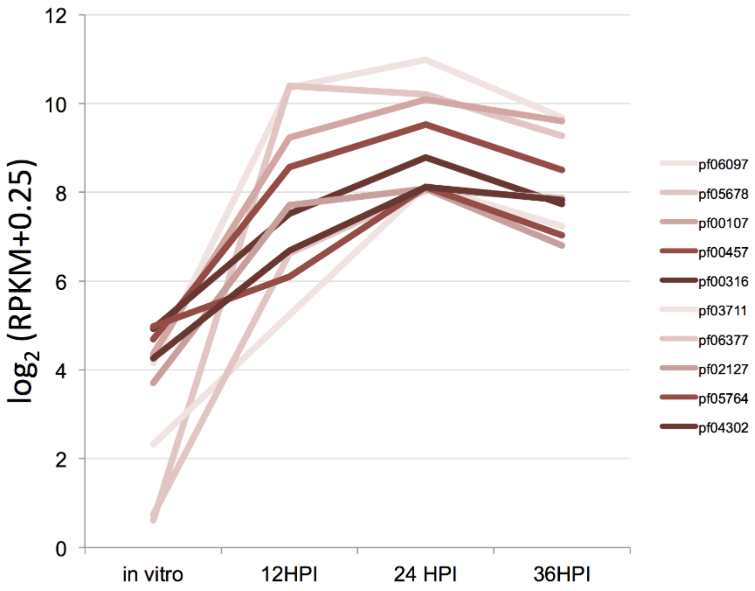

Fig. S4 Transcriptomic profiles of transporter genes in *Pseudozyma flocculosa* during the tripartite interaction *P. flocculosa-Blumeria graminis* f.sp. *hordei-Hordeum vulgare.*

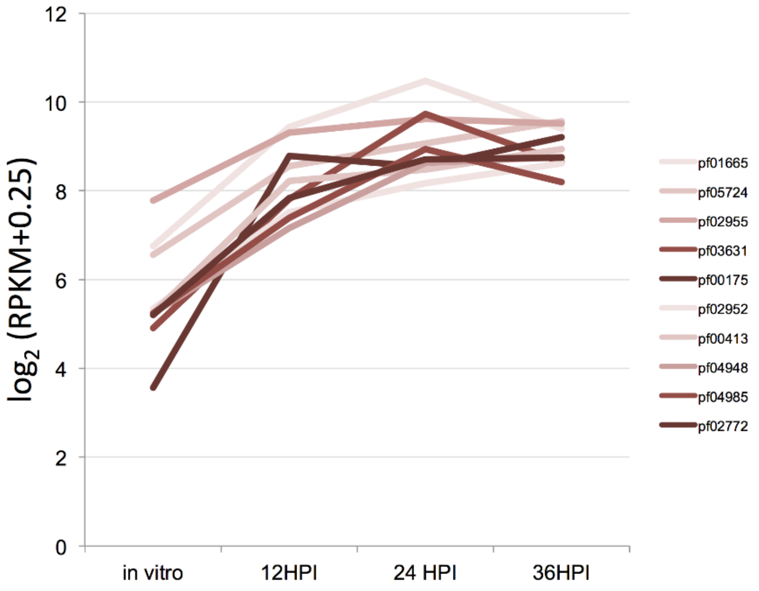

Fig. S5 Gene ontology (GO) enrichment of *Blumeria graminis* differentially expressed genes during the tripartite interaction.

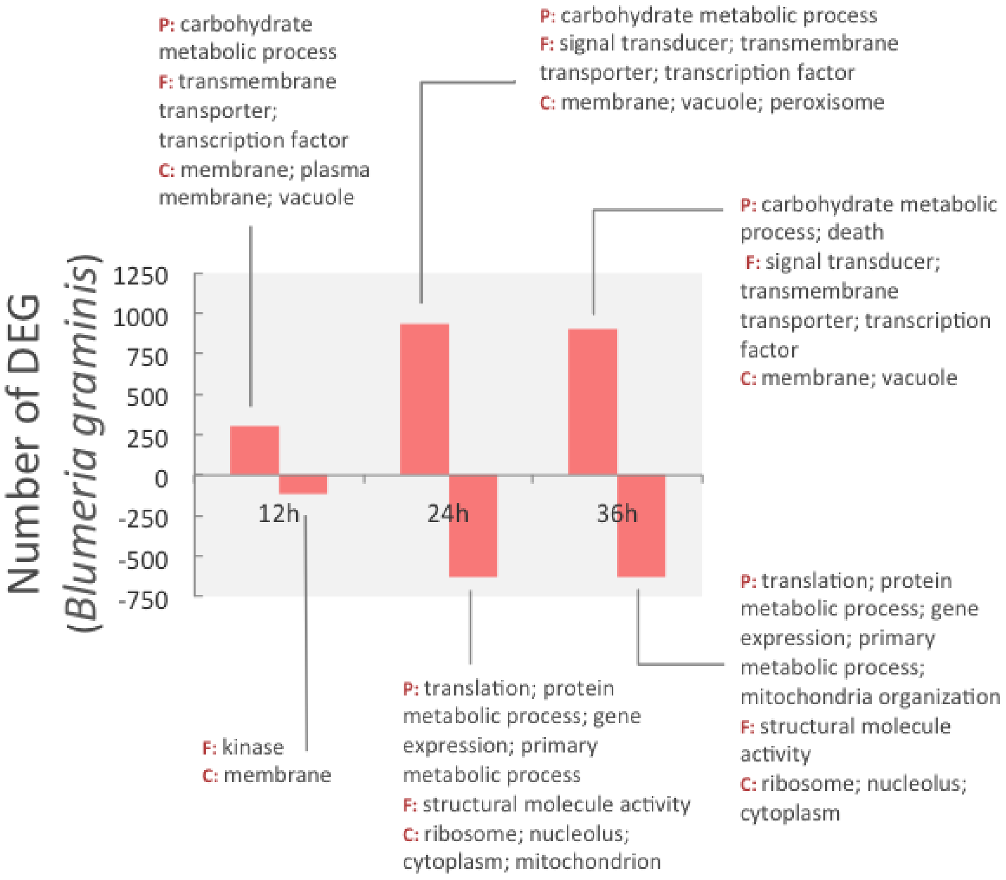

Fig. S6 Transcriptomic profiles of transporter genes in *Blumeria graminis* during the tripartite interaction *Pseudozyma flocculosa-B. graminis* f.sp. *hordei-Hordeum vulgare.*

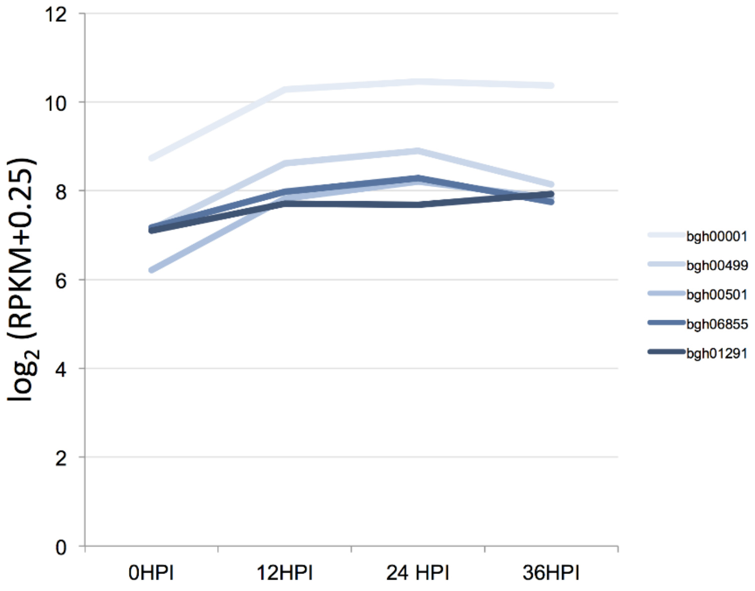

Fig. S7 Transcriptomic profiles of conidia- and hyphae-specific CSEPs in *Blumeria graminis* during the tripartite interaction *Pseudozyma flocculosa-B. graminis* f.sp. *hordei-Hordeum vulgare.*

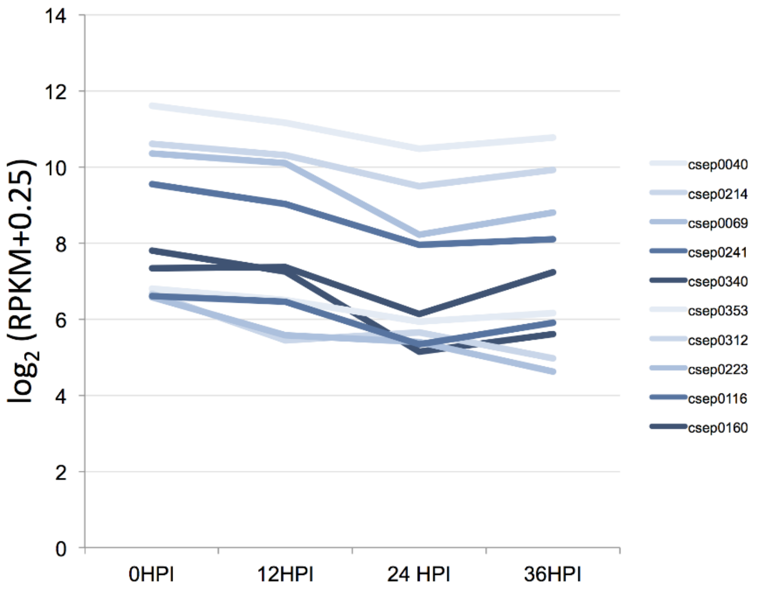

Fig. S8 Transcriptomic profiles of haustoria-specific CSEPs in *Blumeria graminis* during the tripartite interaction *Pseudozyma flocculosa-B. graminis* f.sp. *hordei-Hordeum vulgare.*

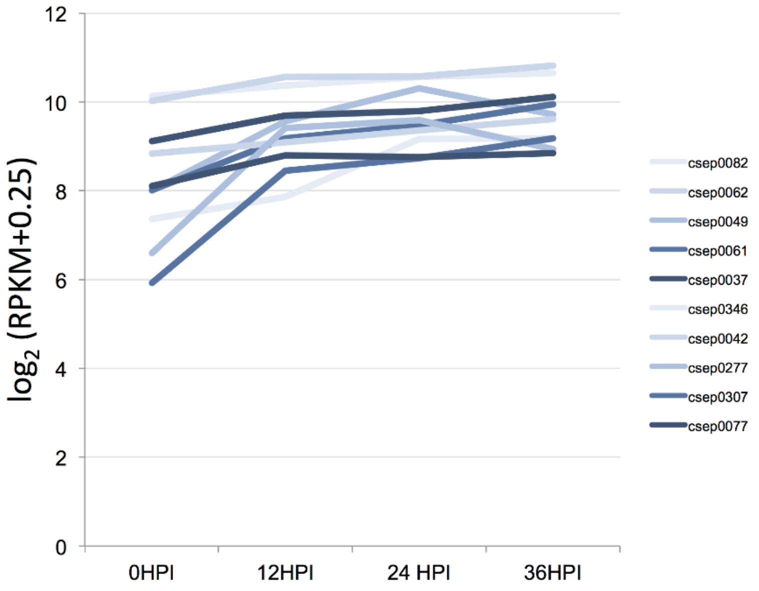

Fig. S9 Gene ontology (GO) enrichment of *Hordeum vulgare* differentially expressed genes during the tripartite interaction.

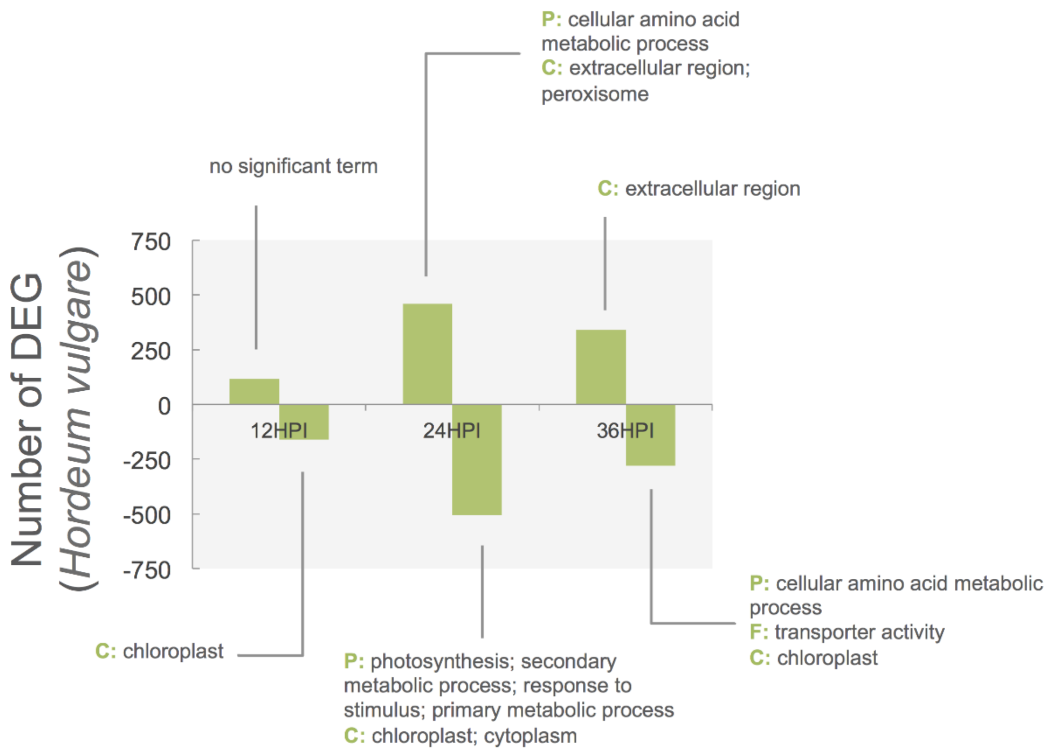

Fig. S10 Transcriptomic profiles of photosynthesis-related genes in *Hordeum vulgare* during the tripartite interaction *Pseudozyma flocculosa-Blumeria graminis* f.sp. *hordei-H. vulgare.*

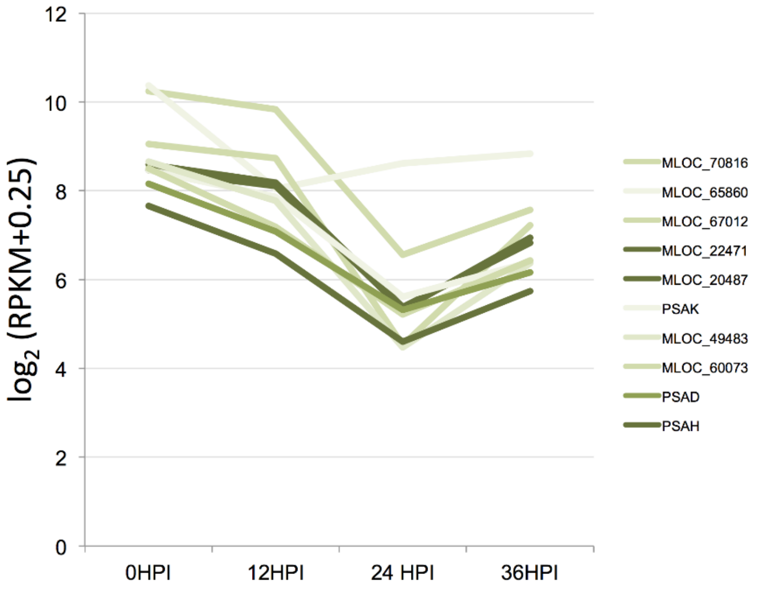

Fig. S11 Transcriptomic profiles of the most expressed genes in *Hordeum vulgare* during the tripartite interaction *Pseudozyma flocculosa-Blumeria graminis* f.sp. *hordei-H. vulgare.*

A

Id	Description	0HPI	12HPI	24HPI	36HPI
***H. vulgare* genes the most expressed**					
MLOC_70346	Pin1-like protease inhibitor	1594	12189	31766	40706
MLOC_52081	PR-1a pathogenesis related protein, Hv-1a	11201	13548	7480	9974
MLOC_43759	Bsi1 protease inhibitor	2346	4354	9706	10728
MLOC_79868	PR4 pathogenesis related protein	4263	5795	6140	9720
MLOC_48531	PR1b pathogenesis related protein	2724	6647	5099	7180
MLOC_71416	BBI-type protease inhibitor	1360	3116	6262	7854
MLOC_80476	BBI-type protease inhibitor	1358	3314	5905	7405
MLOC_17220	glutathione S-transferase	1164	3574	5170	5743
MLOC_71242	PR17 pathogenesis related protein	673	3257	5190	5394
MLOC_65311	Chitinase	1467	2761	3360	5654

B

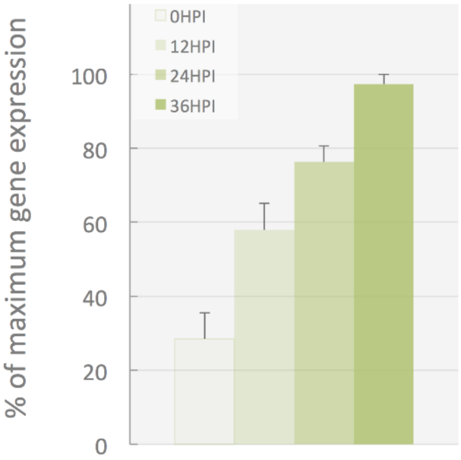

Supplement: Supplementary file 1 — Fig. S1 Transcriptomic profiles of genes associated with flocculosin biosynthesis in Pseudozyma flocculosa during the tripartite interaction P. flocculosa–Blumeria graminis f.sp. hordei–Hordeum vulgare. Fig. S2 Transcriptomic profiles of the most expressed CSEPs genes specific to Pseudozyma flocculosa during the tripartite interaction P. flocculosa–Blumeria graminis f.sp. hordei–Hordeum vulgare. Fig. S3 Transcriptomic profiles of secreted lytic enzyme genes in Pseudozyma flocculosa during the tripartite interaction P. flocculosa–Blumeria graminis f.sp. hordei–Hordeum vulgare. Fig. S4 Transcriptomic profiles of transporter genes in Pseudozyma flocculosa during the tripartite interaction P. flocculosa–Blumeria graminis f.sp. hordei–Hordeum vulgare. Fig. S5 Gene ontology (GO) enrichment of Blumeria graminis differentially expressed genes during the tripartite interaction. Fig. S6 Transcriptomic profiles of transporter genes in Blumeria graminis during the tripartite interaction Pseudozyma flocculosa–B. graminis f.sp. hordei–Hordeum vulgare. Fig. S7 Transcriptomic profiles of conidia‐ and hyphae‐specific CSEPs in Blumeria graminis during the tripartite interaction Pseudozyma flocculosa–B. graminis f.sp. hordei–Hordeum vulgare. Fig. S8 Transcriptomic profiles of haustoria‐specific CSEPs in Blumeria graminis during the tripartite interaction Pseudozyma flocculosa‐B. graminis f.sp. hordei–Hordeum vulgare. Fig. S9 Gene ontology (GO) enrichment of Hordeum vulgare differentially expressed genes during the tripartite interaction. Fig. S10 Transcriptomic profiles of photosynthesis‐related genes in Hordeum vulgareduring the tripartite interaction Pseudozyma flocculosa–Blumeria graminis f.sp. hordei–H. vulgare. Fig. S11 Transcriptomic profiles of the most expressed genes in Hordeum vulgare during the tripartite interaction Pseudozyma flocculosa–Blumeria graminis f.sp. hordei–H. vulgare. [file NPH-217-713-s001.pdf]
